# Mechanisms of Mechanical Stress-Induced Vascular Remodeling via the Lactate-PKM2 Axis and Implications for Microgravity Adaptation

**DOI:** 10.3390/ijms27073298

**Published:** 2026-04-05

**Authors:** Na Li, Ling Liu, Dong Wang, Jing Wang, Yateng Tie, Xi Li, Jiaxiang Li, Yuan Gao, Changbin Yang, Yongchun Wang

**Affiliations:** 1 Department of Aerospace Medical Training, School of Aerospace Medicine, Air Force Medical University, Xi’an 710032, China; yumuhanbo@fmmu.edu.cn (N.L.); wd2460123079@163.com (D.W.); yatengt@163.com (Y.T.); lixi1908@fmmu.edu.cn (X.L.); sch5825l@163.com (J.L.); gaoyuan1109@hotmail.com (Y.G.); 2 Military Medical Innovation Center, Air Force Medical University, Xi’an 710032, China; 18966729388@163.com; 3 Department of Aerospace Ergonomics, School of Aerospace Medicine, Air Force Medical University, Xi’an 710032, China; wangkelu_123@163.com

**Keywords:** mechanical stretch, HASMCs, metabolic reprogramming, sodium lactate, PKM2 lactylation

## Abstract

Vascular remodeling driven by the phenotypic switching of vascular smooth muscle cells (VSMCs) poses a significant health risk to astronauts during long-duration spaceflight. While the morphological and molecular changes are well recognized, the underlying metabolic drivers and potential translational countermeasures remain elusive. To investigate the metabolic determinants of VSMCs phenotypic switching, human aortic smooth muscle cells (HASMCs) were subjected to cyclic mechanical stretch, an in vitro model offering indirect mechanistic insights into mechanical loading conditions relevant to spaceflight-associated hemodynamic alterations. An integrated approach combining quantitative proteomics, flux analysis (Seahorse), and functional assays (cell cycle, wound healing, transwell) was used to characterize the accompanying metabolic and phenotypic alterations. Molecular mechanisms were assessed using immunoprecipitation, protein crosslinking, and immunofluorescence. Mechanical stretch triggered a contractile-to-synthetic phenotypic switch in HASMCs, accompanied by a shift from oxidative phosphorylation to aerobic glycolysis. Pyruvate kinase M2 (PKM2) was identified as a central metabolic regulator of this process, its silencing reversed the pro-synthetic phenotype. Notably, lactate, a glycolytic product, was found to exert a self-limiting feedback signal. Exogenous lactate suppressed the synthetic switch in associated with increased PKM2 lactylation. Further analysis indicated that PKM2 lactylation was associated with enhanced stability of its active tetrameric conformation, which was associated with a metabolic shift toward oxidative phosphorylation and restored expression of contractile markers. Although specific lactylation sites on PKM2 were not identified in this study, and direct causality between lactylation and tetramerization remains to be established, these findings identify a previously unrecognized association. This study reveals a novel metabolic regulatory mechanism in which lactate correlates with the suppression of synthetic switching of VSMCs, linked to PKM2 lactylation and tetramer stabilization. The observed lactate-PKM2 axis represents a candidate metabolic node associated with VSMCs phenotype regulation and offers a potential therapeutic target for modulating vascular remodeling. Upon direct validation under relevant conditions in future studies, this mechanism may inform the development of novel therapeutic strategies for managing vascular adaptation during long-duration spaceflight and other aerospace-related physiological challenges.

## 1. Introduction

Spaceflight poses significant risks to cardiovascular health, with microgravity, radiation, and confinement contributing to vascular dysfunction, which increases the risk of vascular disease in astronauts and is considered a key factor limiting the development of space exploration [1,2,3]. A key manifestation of space-induced vascular remodeling is the increase in carotid artery intima-media thickness [4,5]. This adverse adaptation is primarily driven by the phenotypic switch of vascular smooth muscle cells (VSMCs) from a contractile to a synthetic state [6]. Altered hemodynamics are recognized as the initiating trigger for VSMC phenotypic switching under microgravity [7]. To investigate the mechanisms underlying this pathogenic phenotypic switch, we exposed VSMCs to cyclic mechanical stretch, a well-defined mechanical perturbation that provides indirect mechanistic insight into how altered mechanical loading relevant to microgravity-associated hemodynamic changes may influence VSMC phenotype. While the present study does not directly replicate microgravity conditions, the mechanistic findings offer a framework for future investigations under authentic spaceflight or simulated microgravity settings.

VSMCs within the medial layer primarily display a differentiated, contractile phenotype, characterized by high expression of markers such as α-smooth muscle actin (α-SMA) and smooth muscle protein 22 (SM22). However, in response to injury or stimuli like platelet-derived growth factor-BB (PDGF-BB), VSMCs can undergo phenotypic switching to a dedifferentiated, synthetic state [8]. This transition is characterized by enhanced capacities for proliferation and migration that drive pathological vascular remodeling [9]. Critically, metabolic reprogramming is now recognized as a key driver of phenotypic switching [10]. Whereas contractile VSMCs primarily rely on mitochondrial oxidative phosphorylation for energy, pro-synthetic VSMCs shift toward aerobic glycolysis, accompanied by reduced mitochondrial oxidative activity. Importantly, inhibiting glycolysis has been shown to promote a return to the contractile phenotype, highlighting VSMCs metabolism as a viable target for modulating phenotypic state and mitigating remodeling [11,12].

Many studies have highlighted the critical role of pyruvate kinase M2 (PKM2) in regulating the metabolic state [13,14]. PKM2 catalyzes the transfer of a phosphate group from phosphoenolpyruvate to ADP, generating ATP and pyruvate. Reportedly, PKM2 is subject to complex, allosteric regulation, mainly in three dynamic forms—monomeric, dimeric, and tetrameric—with the dimeric and tetrameric forms exhibiting pyruvate kinase activity [15]. Functionally, the dimeric form, which can translocate to the nucleus, acts primarily as a protein kinase to phosphorylate nuclear substrates and direct glucose-derived carbons toward glycolysis, whereas the tetrameric form is predominantly localized in cytoplasm, functioning as a catalytically active pyruvate kinase and directing glycolytic flux toward the mitochondrial respiratory chain [16,17,18,19]. The transition between these states is precisely regulated by post-translational modifications (PTMs), such as SUMOylation and citrullination, which fine-tune PKM2’s activity and metabolic role [20,21]. Recently, lysine lactylation has emerged as a novel PTM directly induced by lactate [22]. Given that lactate is both a product of PKM2-driven glycolysis and a potential signaling molecule, a critical question arises: could lactate, through modifying PKM2, participate in the phenotypic switching of VSMCs in response to pathological stimuli? In this study, by employing cyclic mechanical stretch to induce VSMCs phenotypic switching, we investigated the association between PKM2 lactylation and VSMC phenotypic regulation. Our findings provide a targeted framework for understanding and potentially intervening in the vascular remodeling driven by VSMCs phenotypic switching under mechanical forces, with potential relevance to conditions such as microgravity that warrant further investigation.

## 2. Results

### 2.1. Cyclic Stretch Induces a Synthetic Phenotype in HASMCs

**Figure 1:**
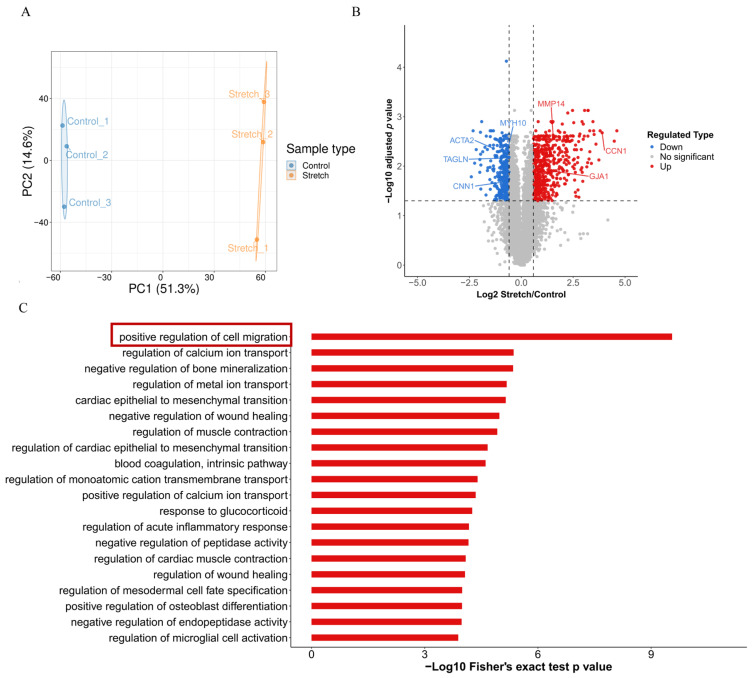
Proteomic changes in HASMCs induced by cyclic mechanical stretch. (**A**) Principal component analysis of the quantified proteome. HASMCs were subjected to cyclic mechanical stretch for 96 h, or maintained under static conditions (control). *n* = 3 biological replicates per group. Protein lysates were analyzed by LC-MS/MS. (**B**) Volcano plot of differentially expressed proteins. Red and blue dots indicate significantly upregulated and downregulated proteins, respectively; gray dots represent non-significant changes. Key proteins associated with phenotypic switching are labeled: ACTA2 (α-SMA), TAGLN (SM22), CNN1 (Calponin 1), MYH10 (Myosin Heavy Chain 10), CCN1, MMP14, and GJA1 (Connexin 43). (**C**) Significantly enriched Gene Ontology (GO) biological process terms among the upregulated proteins. The red box highlights the pathways associated with VSMCs migration.

To investigate the effects of pathological mechanical stress on phenotypic switching in VSMCs, we cultured human aortic smooth muscle cells (HASMCs) on BioFlex plates and exposed them to cyclic mechanical stretch, with cells cultivated under stationary conditions serving as the control. Quantitative proteomic profiling was then employed to characterize the global cellular response to mechanical stretch. Principal component analysis confirmed high reproducibility among replicates within each group (Figure 1A). In total, 1382 differentially expressed proteins (DEPs) were identified (adjusted *p* < 0.05), consisting of 792 upregulated and 590 downregulated proteins (Table S1). Consistent with a phenotypic switch, the expression of key contractile markers, including α-SMA, SM22, calponin 1, and myosin heavy chain 10 (MYH10), was significantly decreased (Figure 1B). Conversely, markers associated with a synthetic and migratory phenotype, such as cellular communication network factor 1 (CCN1) and matrix metalloproteinase 14 (MMP14), were significantly elevated (Figure 1B). Furthermore, we observed an upregulation of connexin 43 (GJA1), which aligns with previous reports showing its involvement in VSMCs proliferation and migration (Figure 1B) [23]. This notion was further supported by pathway enrichment analysis. Gene Ontology (GO) analysis of biological processes revealed significant enrichment in terms such as “positive regulation of cell migration” (Figure 1C). Together, these data demonstrate that cyclic mechanical stretch induces a pro-synthetic phenotypic switch in HASMCs, consistent with vascular remodeling events observed under altered mechanical loading conditions relevant to microgravity.

### 2.2. Cyclic Stretch Promotes a Synthetic Phenotype and Glycolytic Switch in HASMCs

**Figure 2:**
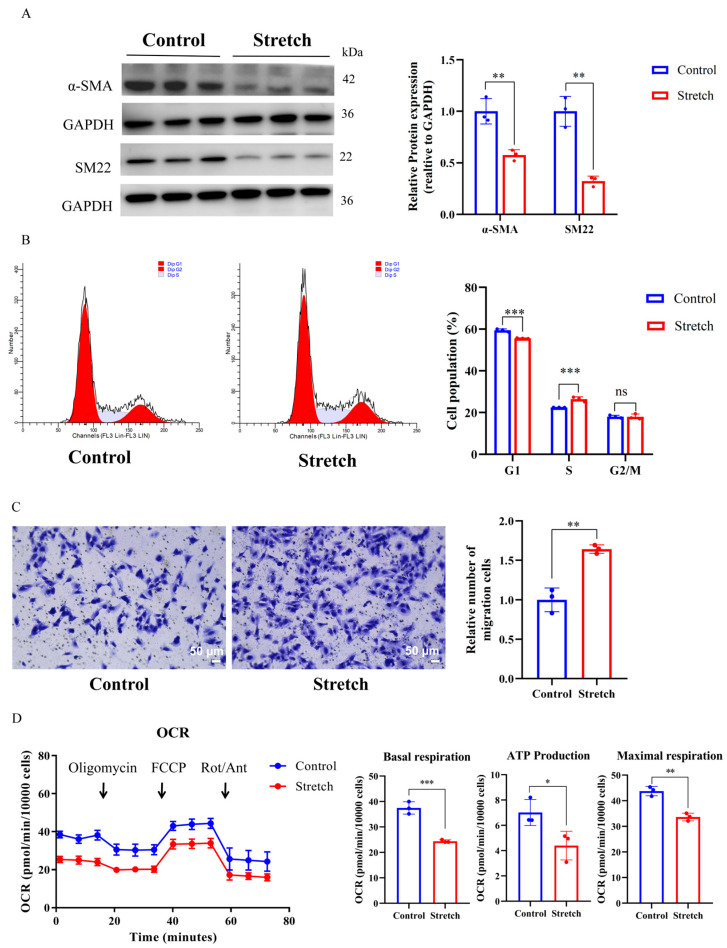
Cyclic mechanical stretch induces a pro-synthetic phenotype and suppresses mitochondrial respiration in HASMCs (**A**) Representative Western blots (left) and quantification (right) of the expression of SM22 and α-SMA in HASMCs after 96 h of mechanical stretch. GAPDH served as an internal control. Data are presented as relative fold change vs. control. (**B**) Representative flow cytometry profiles (left) and quantification (right) of cell cycle distribution after 96 h of mechanical stretch. Cells were stained with propidium iodide (PI). (**C**) Representative images (left) and quantification (right) of transwell migration assay in HASMCs after 96 h of mechanical stretch. Migrated cells were fixed, stained and quantified. Scale bar = 50 µm. (**D**) Oxygen consumption rate (OCR) traces (left) in HASMCs after 96 h of mechanical stretch, measured using an XF96 extracellular flux analyzer. Arrows indicate sequential injections. Quantification (right) of basal respiration, maximal respiration, and ATP production. For all panels, *n* = 3 biological replicates per group. Data are presented as mean ± SD, ns, not significant, * *p* < 0.05, ** *p* < 0.01, *** *p* < 0.001.

Western blot analysis was subsequently employed to validate the proteomic findings. As anticipated, the expression of contractile markers, including SM22 and α-SMA, was significantly downregulated under cyclic mechanical stretch (α-SMA, *p* < 0.01; SM22, *p* < 0.01, Figure 2A). Consistent with this loss of contractile phenotype, the proliferative and migratory capacities of HASMCs were markedly enhanced, which was evidenced by a significant increase in the proportion of cells in the S phase of the cell cycle (*p* < 0.001; Figure 2B) and an increased number of migrated cells in the transwell assay (*p* < 0.01; Figure 2C) compared to the static control.

To assess the associated metabolic state, we measured the mitochondrial oxidative activity in HASMCs under both mechanical stretch and stationary conditions. Seahorse XF96 analysis revealed that cyclic stretch significantly attenuated the oxygen consumption rate (OCR), as evidenced by marked reductions in basal respiration, maximal respiration, and ATP production (*p* < 0.001, *p* < 0.05, and *p* < 0.01, respectively; Figure 2D). These results indicate a pronounced shift toward aerobic glycolysis and impaired mitochondrial oxidative phosphorylation. Collectively, these findings substantiate that cyclic mechanical stretch induces a synthetic phenotypic switch coupled with glycolytic reprogramming in HASMCs, underscoring a critical interplay between metabolic remodeling and phenotypic transition in VSMCs in response to pathological mechanical stress.

### 2.3. PKM2 Knockdown Reverses Stretch-Induced Phenotypic Switching and Glycolytic Reprogramming in HASMCs

**Figure 3:**
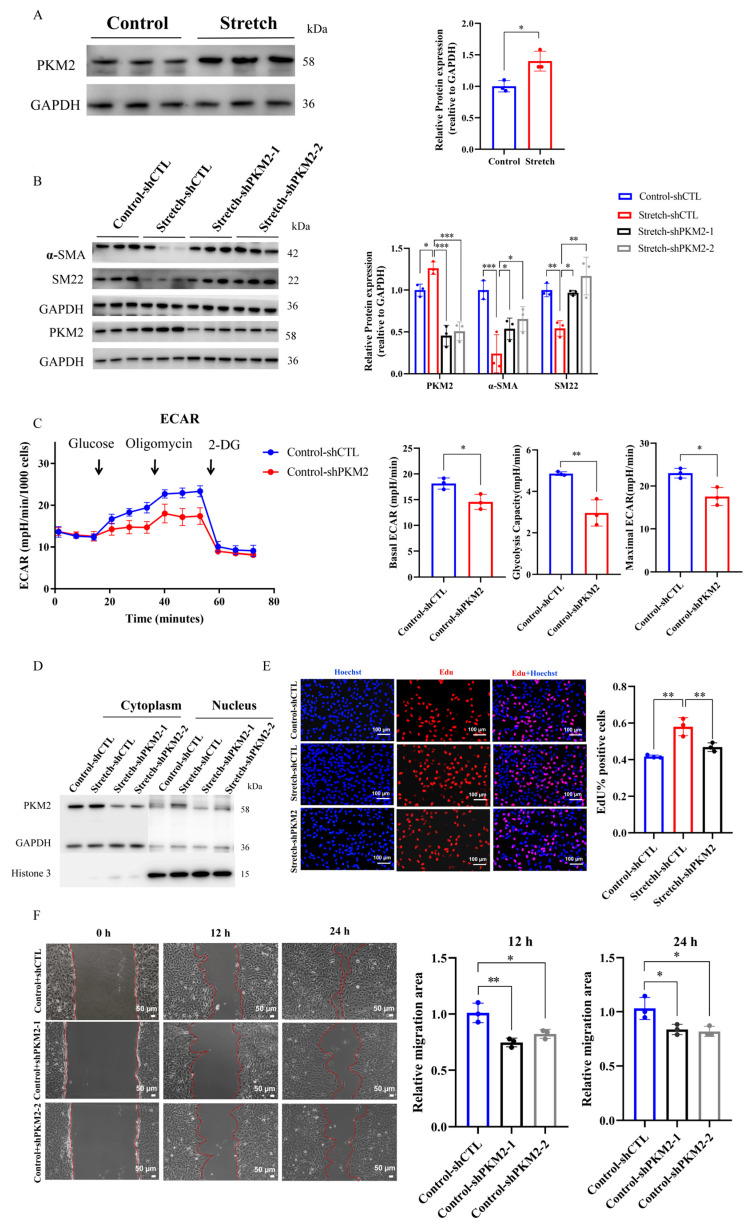
PKM2 silencing reverses the pro-synthetic phenotype of HASMCs induced by cyclic stretch associated with decreased aerobic glycolysis (**A**) ReWestern blotsWestern blots (left) and quantification (right) of dimeric PKM2 expression in HASMCs after 96 h of mechanical stretch. GAPDH served as an internal control. (**B**) Representative western blots (left) and quantification (right) of contractile markers (α-SMA and SM22) in HASMCs transfected with LV-shCTL or LV-shPKM2 after 96 h of mechanical stretch. Data are presented as relative fold change vs. control-shCTL. (**C**) Extracellular acidification rate (ECAR) traces (left) in HASMCs transfected with LV-shCTL or LV-shPKM2 after 96 h of mechanical stretch, measured by an XF96 extracellular flux analyzer. Arrows indicate sequential injections. Quantification (right) of basal ECAR, glycolytic capacity, and maximal ECAR. (**D**) Western blot analysis of dimeric PKM2 in cytoplasmic (left) and nuclear (right) fractions of HASMCs transfected with LV-shCTL or LV-shPKM2 after 96 h of mechanical stretch. GAPDH and Histone H3 served as loading controls for cytoplasmic and nuclear fractions, respectively. (**E**) Representative fluorescence images (left) and quantification (right) of EdU incorporation in HASMCs after 96 h of mechanical stretch. Scale bar = 100 µm. (**F**) Representative images (left) and quantification (right) of wound healing assay in HASMCs after 96 h of mechanical stretch. Images were taken at 0, 12 h, and 24 h after scratching. Scale bar = 50 µm. For all panels, *n* = 3 biological replicates per group. Data are presented as mean ± SD, * *p* < 0.05, ** *p* < 0.01, *** *p* < 0.001. shCTL, HASMCs transfected with LV-NC; shPKM2-1 and shPKM2-2, HASMCs transfected with LV encoding two distinct shRNAs targeting PKM2.

Recent in vitro and in vivo studies indicate that synthetic VSMCs exhibit enhanced aerobic glycolysis and upregulation of dimeric PKM2 compared to their contractile counterparts [19]. To determine whether the levels of dimeric PKM2 are altered by cyclic mechanical stretch, we performed Western blot analysis and observed a significant increase in dimeric PKM2 levels in HASMCs exposed to mechanical stretch (*p* < 0.05; Figure 3A). To investigate the functional role of PKM2, we generated PKM2-knockdown HASMCs using lentivirus-delivered shRNA (LV-shPKM2). LV-shPKM2 transfection effectively suppressed PKM2 expression (*p* < 0.001; Figure 3B) and significantly increased expression of contractile markers (α-SMA, *p* < 0.05; SM22, *p* < 0.05; Figure 3B). Additionally, PKM2 knockdown reduced aerobic glycolysis, as shown by significant decreases in extracellular acidification rate (ECAR), maximal ECAR and glycolytic capacity (*p* < 0.05, *p* < 0.01, and *p* < 0.05, respectively, Figure 3C), consistent with reduced glycolytic activity.

Since dimeric PKM2 can translocate to the nucleus and promote cell-cycle progression, we examined its subcellular localization. In control HASMCs, dimeric PKM2 was predominantly cytoplasmic; under cyclic stretch, it showed increased nuclear accumulation, whereas PKM2 knockdown attenuated this translocation (Figure 3D). Consistent with these findings, 5-ethynyl-2′-deoxyuridine (EdU) incorporation and wound healing assays confirmed that PKM2 silencing significantly suppressed stretch-induced proliferation (*p* < 0.01; Figure 3E) and migration (*p* < 0.05 at 12 h and 24 h; Figure 3F). Together, these results demonstrate that PKM2 knockdown mitigates the pro-synthetic phenotypic switch in HASMCs under cyclic mechanical stretch, accompanied by restored contractile marker expression, reduced glycolytic activity, and suppressed proliferation and migration. These findings identify PKM2 as a critical node linking mechanical stress to metabolic and phenotypic reprogramming in VSMCs.

### 2.4. Lactate Infusion Maintains Contractile Phenotype by Inducing PKM2 Lactylation and Tetramerization

**Figure 4:**
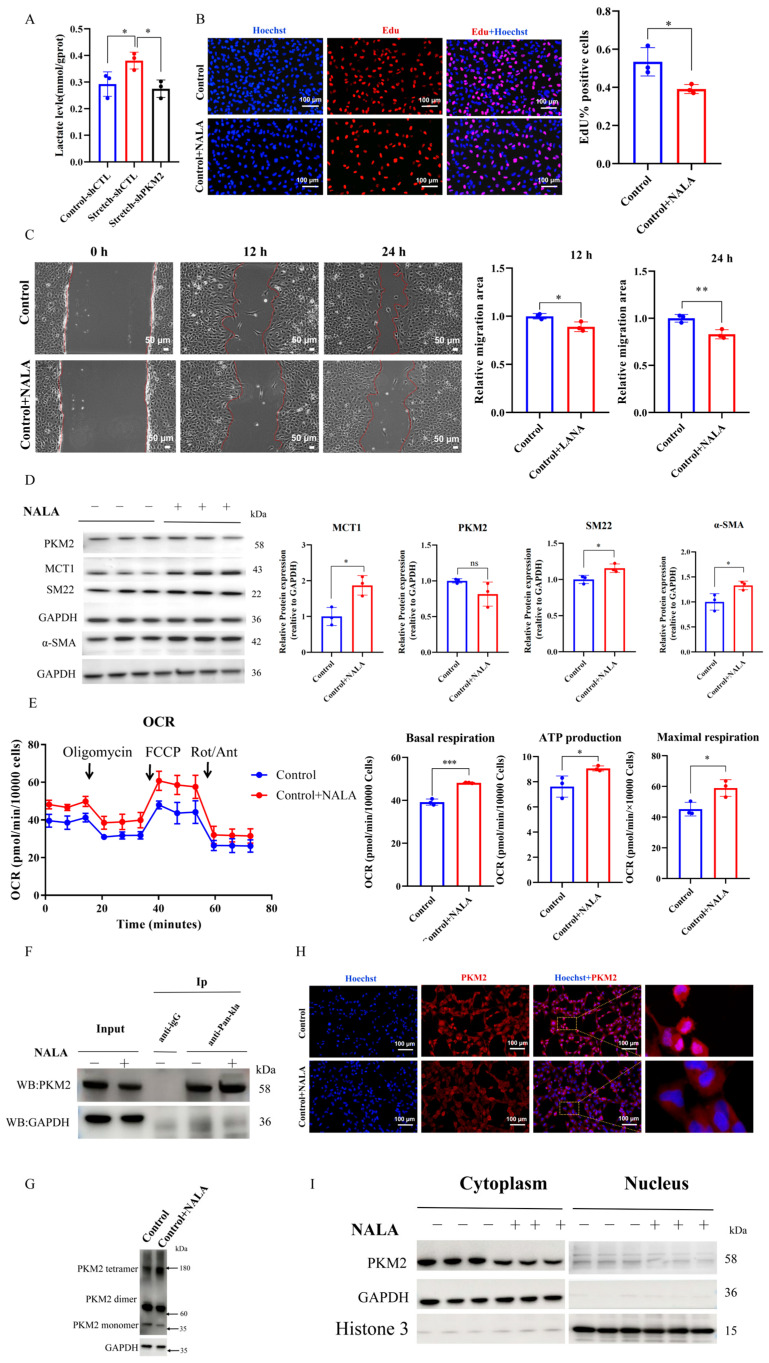
Lactate promotes a contractile phenotype by enhancing PKM2 lactylation. (**A**) Lactate concentration in HASMCs transfected with LV-shCTL or LV-shPKM2 after 96 h of mechanical stretch. (**B**) ReWestern blotsfluorescence images (left) and quantification (right) of EdU incorporation in HASMCs after 48 h of treatment with or without NALA (20 mM). Scale bar = 100 µm. (**C**) Representative images (left) and quantification (right) of wound healing assay in HASMCs after 48 h of treatment with or without NALA (20 mM). Images were taken at 0, 12 h, and 24 h after scratching. Scale bar = 50 µm. (**D**) Representative Western blots (left) and quantification (right) of contractile markers (SM22, α-SMA) and MCT1 in lysates of HASMCs after 48 h of treatment with or without NALA (20 mM). GAPDH served as an internal control. (**E**) OCR traces (left) in HASMCs after 48 h of treatment with or without NALA (20 mM), measured using an XF96 extracellular flux analyzer. Arrows indicate sequential injections. Quantification (right) of basal respiration, maximal respiration, and ATP production. (**F**) Immunoprecipitation assay of PKM2 lactylation in lysates of HASMCs after 48 h of treatment with or without NALA (20 mM). Lysates were immunoprecipitated with pan-Kla antibody, and the precipitates were immunoblotted with anti-PKM2 antibody using a light chain-specific secondary antibody to minimize IgG interference. (**G**) Representative western blots of dimeric and tetrameric PKM2 in lysates of HASMCs after 48 h of treatment with or without NALA (20 mM). Cells were crosslinked with disuccinimidyl suberate (DSS) prior to lysis. GAPDH served as an internal control. (**H**) Representative confocal fluorescence images of PKM2 (red) in HASMCs after 48 h of treatment with or without NALA (20 mM). Nuclei were stained with DAPI (blue). Scale bar = 100 µm. (**I**) Western blot analysis of PKM2 in the cytoplasmic and nuclear fractions of HASMCs after 48 h of treatment with or without NALA (20 mM). GAPDH and Histone H3 served as loading controls for cytoplasmic and nuclear fractions, respectively. For all panels, *n* = 3 biological replicates per group. Data are presented as mean ± SD, ns, not significant, * *p* < 0.05, ** *p* < 0.01, *** *p* < 0.001. NALA, Sodium Lactate.

As PKM2 is a key glycolytic enzyme that drives lactate production, we first measured lactate levels in HASMCs undergoing stretch-induced phenotypic switching. Consistent with the upregulation of dimeric PKM2, lactate concentration was significantly elevated in mechanically stretched HASMCs (*p* < 0.05; Figure 4A) but markedly decreased upon PKM2 knockdown (*p* < 0.05; Figure 4A). To investigate whether lactate itself influences the HASMC phenotype, cells were cultured with or without 20 mM sodium lactate (NALA) for 48 h under static conditions. NALA supplementation significantly reduced the number of proliferating (*p* < 0.05; Figure 4B) and migrating cells (*p* < 0.05 at 12 h, *p* < 0.01 at 24 h; Figure 4C). Furthermore, lactate treatment increased the expression of contractile markers (α-SMA and SM22, both *p* < 0.05; Figure 4D) and upregulated monocarboxylate transporter 1 (MCT1, *p* < 0.05; Figure 4D), which facilitates lactate uptake. These effects were similar to those observed in PKM2-knockdown cells, suggesting that lactate may promote a contractile phenotype. To explore the underlying mechanism, we assessed whether lactate reprograms cellular metabolism. Seahorse XF96 analysis revealed that lactate treatment significantly increased OCR (basal respiration, *p* < 0.001; ATP production, *p* < 0.05; maximal respiration, *p* < 0.05; Figure 4E), consistent with a metabolic shift from aerobic glycolysis toward oxidative phosphorylation.

Considering that lactate serves as a functional substrate for lysine lactylation and PKM2 is susceptible to conformational regulation via PTM, we examined whether lactate induces PKM2 lactylation. Co-immunoprecipitation using a pan-lactylation antibody revealed elevated PKM2 lactylation in cells exposed to lactate (Figure 4F). This modification was associated with a decrease in dimeric PKM2 and a concomitant increase in its tetrameric form (Figure 4G) [17]. Moreover, lactate treatment reduced nuclear translocation of PKM2, as shown by immunofluorescence and Western blotting (Figure 4H,I). Taken together, these findings suggest that lactate may act as a feedback regulator that induces PKM2 lactylation, which is associated with increased tetramerization and reduced nuclear localization, thereby potentially contributing to the maintenance of a contractile phenotype in HASMCs. However, the specific lactylation sites on PKM2 and the direct causal relationship between lactylation and tetramerization remain to be determined in future studies.

### 2.5. Lactate Infusion Reverses Stretch-Induced Pro-Synthetic Phenotype by Promoting PKM2 Tetramerization

**Figure 5:**
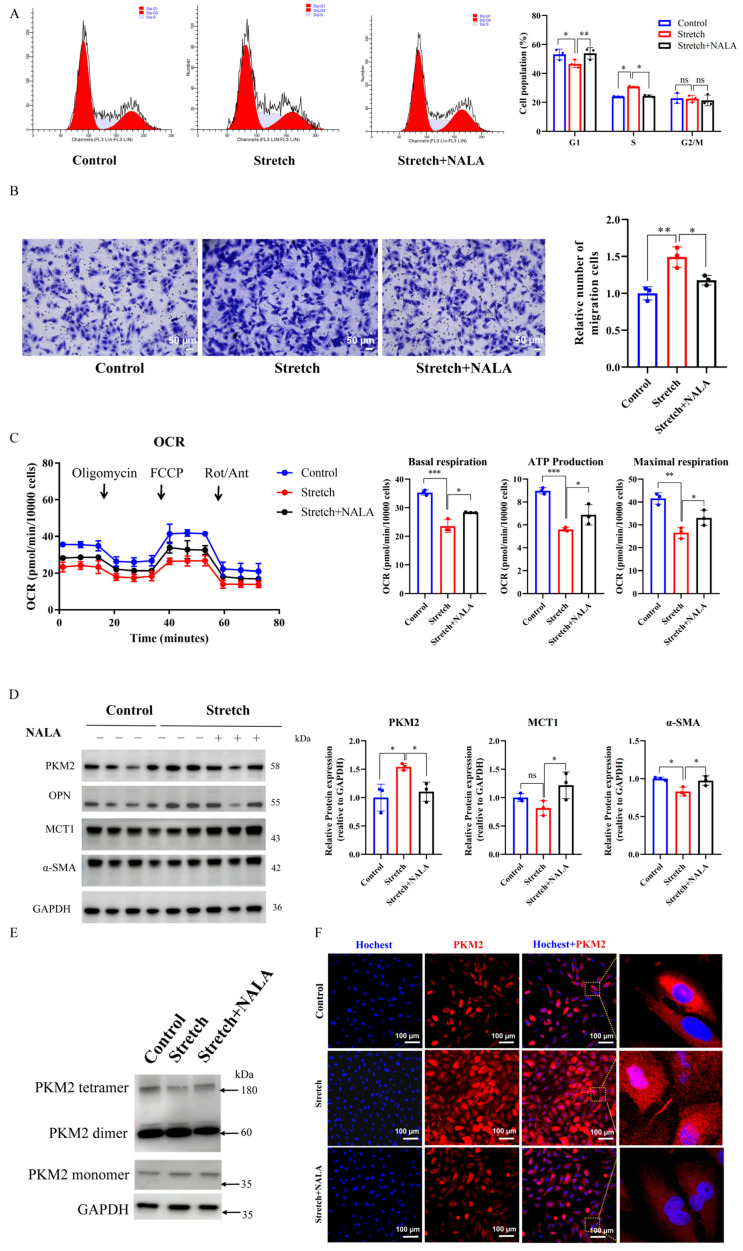
Lactate reverses the pro-synthetic phenotype induced by mechanical stretch. (**A**) ReWestern blotsflow cytometry profiles (left) and quantification (right) of cell cycle distribution after 96 h of mechanical stretch with or without NALA (20 mM). (**B**) Representative images (left) and quantification (right) of transwell migration assay in HASMCs after 96 h of mechanical stretch with or without NALA (20 mM). Migrated cells were fixed, stained and quantified. Scale bar = 50 µm. (**C**) OCR traces (left) in HASMCs after 96 h of mechanical stretch with or without NALA (20 mM), measured using an XF96 extracellular flux analyzer. Arrows indicate sequential injections. Quantification (right) of basal respiration, maximal respiration, and ATP production. (**D**) Representative Western blots (left) and quantification (right) of contraction markers (α-SMA, SM22) in HASMCs after 96 h of mechanical stretch with or without NALA (20 mM). GAPDH served as an internal control. Data are presented as relative fold change vs. control. (**E**) Representative western blots of dimeric and tetrameric PKM2 in lysate of HASMCs after 96 h of mechanical stretch with or without NALA (20 mM). Cells were crosslinked with DSS prior to lysis. GAPDH served as an internal control. (**F**) Representative confocal fluorescence of PKM2 (red) in HASMCs exposed to stretch for 96 h with or without NALA. Nuclei were stained with DAPI (blue). Scale bar = 100 µm. For all panels, *n* = 3 biological replicates per group. Data are presented as mean ± SD, ns, not significant, * *p* < 0.05, ** *p* < 0.01, *** *p* < 0.001.

We next investigated whether lactate infusion modulates the pro-synthetic phenotype induced by mechanical stretch, with a focus on PKM2 oligomerization. HASMCs were cultured in DMEM supplemented with or without NALA and subjected to cyclic stretch. Lactate treatment significantly attenuated the mechanical stretch-induced increase in the proportion of S phase cells (*p* < 0.05; Figure 5A) and cell migration (*p* < 0.05; Figure 5B), as assessed by propidium iodide (PI) staining and transwell assays, respectively. Of note, Seahorse XF96 analysis revealed that lactate significantly counteracted the stretch-induced decline in OCR, improving basal respiration, ATP production and maximal respiration (each *p* < 0.05; Figure 5C).

Consistent with these functional observations, lactate opposed the stretch-induced changes, significantly downregulated the expression of dimeric PKM2 (*p* < 0.05; Figure 5D) and upregulated the contractile marker α-SMA (*p* < 0.05; Figure 5D). Concurrently, lactate elevated the expression of MCT1 (*p* < 0.05; Figure 5D). At the molecular level, lactate reduced the stretch-induced elevation of dimeric PKM2 and promoted its transition to the tetrameric form (Figure 5E), accompanied by diminished nuclear translocation of PKM2 (Figure 5F). Together, these findings suggest that lactate-induced PKM2 lactylation is associated with the prevention of pro-synthetic phenotypic switching under mechanical stretch, potentially by shifting PKM2 from a glycolysis-promoting dimer to a tetramer that favors oxidative phosphorylation.

## 3. Discussion

In this study, we demonstrate that cyclic mechanical stretch, a canonical inducer of pathological vascular remodeling, triggers a synthetic phenotype in HASMCs along with a metabolic shift toward glycolysis. This is consistent with key features of VSMCs dysfunction in mechanical stretch models and aligns with the vascular structural remodeling observed under altered mechanical environments. We identify the glycolytic enzyme PKM2 as a central regulator of this process: its dimeric form is upregulated by mechanical stress, and its knockdown reverses the pro-synthetic switch. Notably, we suggest a potential self-limiting feedback circuit in which lactate, a product of PKM2 driven glycolysis, is associated with suppression of the very phenotype that generates it. Mechanistically, lactate is associated with PKM2 lactylation, which may stabilize the enzyme in its tetrameric state, although the specific modification sites remain to be identified. This conformational shift correlates with redirected metabolic flux toward oxidative phosphorylation and correlates with attenuation of the synthetic phenotype. These findings reveal PKM2 lactylation as a critical metabolic checkpoint governing VSMC plasticity in response to mechanical stress. Given that VSMC phenotypic switching is a convergent endpoint in mechanical stress-induced vascular remodeling, targeting the lactate-PKM2 axis may represent a potential translational strategy to mitigate vascular remodeling under conditions of altered mechanical stress.

The cyclic stretch applied in this study (1 Hz, 15% elongation) is a well-established biomechanical model that effectively induces a pro-synthetic phenotype in VSMCs, mimicking the phenotypic switching observed in various vascular pathologies [24]. The frequency of 1 Hz approximates the human resting heart rate, and 15% strain corresponds to the upper physiological limit of circumferential strain in arteries during the cardiac cycle, parameters widely used to modulate VSMC biology [25,26,27]. Importantly, this in vitro model provides indirect mechanistic insight rather than direct simulation of the complex, systemic hemodynamic environment (such as the cephalad fluid shift) associated with microgravity [28]. Instead, it serves as a controlled and direct mechanical perturbation to induce the pathogenic phenotypic switch, thereby enabling the systematic dissection of the underlying mechanisms. While the phenotypic outcome induced by such cyclic stretch is recognized, the accompanying metabolic reprogramming—a fundamental driver of cellular plasticity—has remained poorly defined. This study focuses on elucidating this critical metabolic layer of the phenotypic switching process.

It is well recognized that PKM2, a key glycolytic enzyme, plays a central role in regulating metabolic reprogramming [14]. Upregulation of PKM2 generally promotes a shift toward aerobic glycolysis, whereas its downregulation can mitigate this transition [19]. In the present study, we generated PKM2 knockdown HASMCs to investigate whether PKM2 silencing could suppress proliferation under mechanical stress. Our results align with previous reports using oxidized low-density lipoprotein (oxLDL) and the PKM2-specific inhibitor shikonin: while oxLDL stimulates VSMC proliferation and migration, shikonin counteracts these effects [29]. Similarly, PKM2 silencing in our model significantly elevated contractile marker expression and inhibited the proliferation and migration of HASMCs under mechanical stress. At the mechanistic level, PKM2 knockdown led to a pronounced decrease in ECAR compared to control cells (LV-shCTL) under the same stress conditions [30]. These findings suggest that PKM2 plays a critical role in the pro-synthetic switch in VSMCs under mechanical stress and that targeting PKM2-dependent glycolysis may represent a potential strategy to modulate this phenotype.

Strategies targeting PKM2 to regulate glycolysis have been extensively explored in numerous studies [31]. Recent evidence indicates that the catalytic activity of PKM2 is subject to regulation by post-translational modifications (PTMs). Various forms of PTMs—including phosphorylation, methylation, acetylation, oxidation, hydroxylation, succinylation, and glycylation—have been identified at distinct amino acid residues within the PKM2 sequence [18]. For instance, phosphorylation at tyrosine 105 inhibits the formation of active tetrameric PKM2 by interfering with the binding of its cofactor fructose-1,6-bisphosphate [32]. Similarly, oxidation at cysteine 358 or 423 has been shown to reduce pyruvate kinase activity [33]. Furthermore, crotonylation at K305 enhances PKM2 dimerization and promotes VSMC proliferation [34]. In contrast, methylation at arginines 445 and 447 mediated by co-activator-associated arginine methyltransferase 1 (CARM1) facilitates PKM2 tetramer formation, thereby elevating pyruvate kinase activity [35]. More recently, lactate, once considered merely a metabolic byproduct, has emerged as a key signaling molecule that bridges cellular metabolism with functional regulation. Since the initial identification of lysine lactylation in 2019 [36], this post-translational modification has been implicated in a range of pathophysiological processes. For example, lactate was reported to increase oxidative phosphorylation (OXPHOS) in macrophages by enhancing PKM2 lactylation at lysine 62. This modification appears to stabilize the enzyme in its tetrameric state, boosts pyruvate kinase activity, limits its nuclear translocation, and facilitates the transition of pro-inflammatory macrophages toward a reparative phenotype [37]. Our findings suggest that lactate exerts a parallel, phenotype-stabilizing effect in VSMCs, underscoring the potential conserved significance of this metabolic feedback loop. Consistent with these findings, our study indicates that lactate is associated with a shift from a pro-synthetic to a pro-contractile phenotype in HASMCs, coinciding with increased PKM2 lactylation. The modification correlates with the transition of PKM2 from a dimeric to a tetrameric state, and with redirected glucose-derived carbon flux toward the respiratory chain, as indicated by an increase in OCR. Notably, while prior evidence suggests lysine 62 of PKM2 as a potential lactylation site in other cell types, the specific modification site (s) were not identified in our current dataset. Collectively, these studies underscore the importance of diverse PTMs in modulating the dynamic equilibrium between PKM2 dimers and tetramers, a balance that critically influences cellular phenotype and fate. While the distinct functions of PKM2 dimers (e.g., protein kinase activity) and tetramers (e.g., ATP production) are well-established in dictating cellular fate [38,39,40], our work introduces lactylation as a critical PTM that may govern this equilibrium in VSMCs in response to biomechanical cues. The association of this axis with phenotypic modulation through lactate supplementation in vitro provides a mechanistic basis for further investigation. Future studies in integrated physiological models (e.g., hindlimb unloading) will be crucial to determine whether targeting PKM2 lactylation can effectively modulate vascular remodeling in response to systemic hemodynamic challenges, such as those encountered under altered gravitational conditions.

Our observation that sodium lactate supplementation upregulates the lactate transporter MCT1 suggests a potential mechanistic link for its action. This aligns with and functionally supports the lactate shuttle theory, which posits that monocarboxylate transporters (MCTs) are essential for shuttling lactate across cellular and tissue compartments [41]. Among MCT isoforms, MCT1 is ubiquitously expressed and facilitates bidirectional lactate transport, enabling it to respond to metabolic demands. In the context of our model, the upregulation of MCT1 may contribute to establishing a feed forward loop that enhances lactate uptake, thereby potentially amplifying the intracellular lactate signal that may contribute to promoting PKM2 lactylation and the subsequent phenotypic shift. Thus, our findings suggest an extension of the lactate shuttle concept beyond metabolic coupling to include a potential self-reinforcing signaling role in the adaptive mechano-metabolic response of VSMCs.

**Figure 6:**
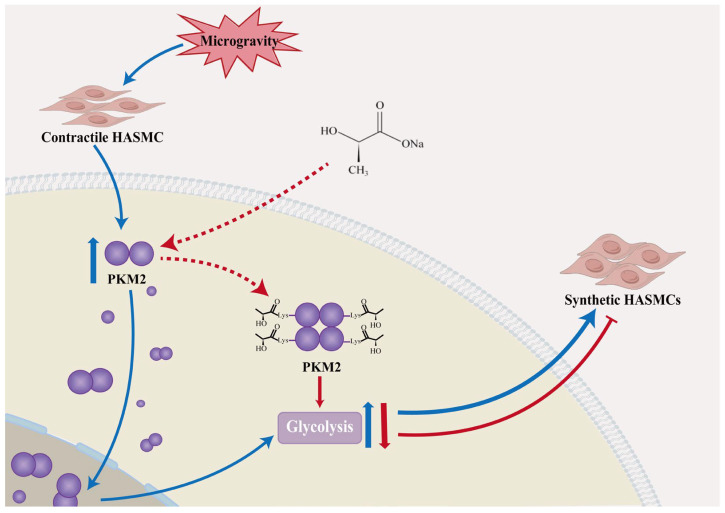
Proposed mechanism of PKM2 lactylation in regulating the phenotypic switch of HASMCs in response to cyclic stress. Under cyclic mechanical stress, HASMCs undergo a transition toward a pro-synthetic phenotype, which is assocaited with metabolic reprogramming and upregulation of dimeric PKM2. Lactate treatment attenuates this switch in association with PKM2 lactylation, potentially stabilizing the enzyme in its active tetrameric conformation. This correlates with a shifts in cellular metabolism from aerobic glycolysis toward oxidative phosphorylation, thereby potentially contributing to the restoration of the contractile phenotype.

In summary, our findings suggest a mechanistic pathway through which cyclic mechanical stress is associated with a synthetic phenotype in HASMCs involving glycolytic reprogramming. We suggest the glycolytic enzyme PKM2 as a potential molecular switch governing this process. A key observation is a potential self-limiting feedback mechanism in which lactate, a glycolytic byproduct, is associated with suppression of the synthetic phenotype, coinciding with PKM2 lactylation and stabilization of its active tetrameric form. This correlates with reroutes metabolic flux from biosynthetic pathways toward oxidative phosphorylation, thereby potentially counteracting the initial pro-synthetic trigger, as shown in Figure 6. The lactate-PKM2 regulatory axis we identified may represent a potential target for interventions against pathological vascular remodeling driven by VSMC dysfunction, with potential relevance to mitigating vascular remodeling under conditions of altered mechanical stress.

## 4. Materials and Methods

### 4.1. Reagents

Antibodies and reagents were purchased from the following companies: Anti-SM22 (10234-2-AP, Proteintech, Rosemont, IL, USA), Anti-α-SMA (Proteintech, 14395-1-AP), Anti-GAPDH (Proteintech, 10494-1-AP), Anti-PKM2 (Proteintech, 15822-1-AP), Anti-MCT1 (Proteintech, 20139-1-AP), Anti-Histone 3 (Proteintech, 17168-1-AP), Anti-IgG (Proteintech, 98136-1-RR), HRP-conjugated Goat Anti-Rabbit (Proteintech, SA00001-2), Anti-L-Lactyl Lysine Rabbit mAb (PTM-1401RM, Jingjie Biotechnology Co., Hangzhou, China), Sodium lactate (L7022, Sigma-Aldrich, St. Louis, MO, USA), Cell-Light^TM^ EdU Apollo In Vitro Kit (10310-1, RiboBio, Guangzhou, China), HASMCs (CTCC-001-0577-CM, MeisenCTCC, Hangzhou, China), DMEM/F12 medium (PM150312, Procell, Wuhan, China), fetal bovine serum (FBS) (BS-1101, OPCEL, Hohhot, Inner Mongolia, China), penicillin and streptomycin (C100C5, NCM Biotech, Suzhou, China), RIPA (WB3100, NCM Biotech), BCA (P0010S, Beyotime, Shanghai, China), PVDF membrane (ISEQ00010, Millipore, Burlington, MA, USA), HyperSignal ECL (4AW012-500, 4A BIOTECH, Beijing, China), 6-Well flexible-bottom culture plates (BF-3001C, Flexcell International Corporation, Burlington, NC, USA), Disuccinimidyl suberate (DSS) (A39267, Thermo Scientific, Waltham, MA, USA), Nuclear and Cytoplasmic Protein Extraction Kit (P0028, Beyotime), rProteinA/G Magnetic IP/Co-IP Kit (BK0004-02, ACE, Nanjing, China), PI/RNase (550825, BD Pharmingen, San Jose, CA, USA).

### 4.2. Cell Culture and Transfection

HASMCs were cultured in a humidified atmosphere of 5% CO_2_ at 37 °C and maintained in complete DMEM/F12 medium supplemented with 10% fetal bovine serum (FBS), 1% penicillin, and 1% streptomycin. During the wound healing assay, cells were switched to serum-free DMEM/F12 medium. To generate PKM2-knockdown and corresponding control cells, PKM2-targeting siRNA and negative control (NC) siRNA were designed and packaged into lentiviral vectors by GeneChem Technology Co., Ltd. (Shanghai, China), yielding LV-shPKM2 and LV-shCTL, respectively. The siRNA sequences are provided in the Table S2. For lentiviral transduction, HASMCs were seeded in 6-well plates at a density of 1.5 × 10^5^ cells/mL. When cells reached approximately 30% confluence, they were transduced with LV-shPKM2 or LV-shCTL at a multiplicity of infection (MOI) of 20. After 48 h, by which time confluence had reached about 90%, the cells were harvested and transferred to T25 culture flasks. The medium was then replaced with fresh complete medium containing 3 µg/mL puromycin for selection. Transduced cells were continuously maintained in puromycin-containing medium until stable PKM2-knockdown and negative control HASMC lines were established.

### 4.3. Mechanical Stretch Application

HASMCs, PKM2-KO HASMCs, and NC HASMCs were seeded on flexible silicone membranes at a density of 2 × 10^5^ cells per well. After cell attachment, the cultures were subjected to mechanical stretch using the FX-6000T (Tension) (Flexcell International Corporation) controlled by FlexSoft® FX-6000^TM^ V1.0 (Flexcell International Corporation). This system enables precise cyclic stretching through regulated vacuum application beneath the elastomer membranes. A uniform elongation of 15% with 1 Hz was applied continuously for 96 h.

### 4.4. Protein Sample Preparation and Proteomic Analysis

Protein extraction, digestion, and liquid chromatography-tandem mass spectrometry (LC-MS/MS) analysis were conducted by Jingjie Biotechnology Co., Ltd. (Hangzhou, China). Briefly, proteins extracted from HASMCs under simulated stretch or static control conditions were reduced, alkylated, and digested with trypsin. The resulting peptides were separated by nanoflow ultra-high performance liquid chromatography (UHPLC) and analyzed on a timsTOF Pro mass spectrometer operated in data-independent acquisition (DIA) mode. Raw data were processed using Spectronaut software 17.0 (Biognosys AG, Schlieren, Switzerland) with the [e.g., UniProt Human] database for protein identification and quantification. Differential expression analysis was performed using a significance threshold of |log_2_(fold change)| > 1.5 and adjust *p* value < 0.05.

### 4.5. EdU Incorporation Assay

Cell proliferation was assessed using the Cell-Light™ EdU Apollo In Vitro Kit. Following 96 h exposure to mechanical stretch or 48 h supplementation with sodium lactate (NALA, 20 mM), cells in 6-well plates were incubated with 1 mL of fresh medium containing an equal volume of 1× EdU working solution (50 µM) for 2 h. Subsequently, the cells were fixed with 4% paraformaldehyde for 15 min and stained with Apollo solution for 30 min, followed by Hoechst 33342 for 10 min at room temperature. Fluorescence images were acquired using a Nikon ECLIPSE Ti2 inverted microscope (Nikon Instruments Inc., Tokyo, Japan) and processed with NIS-Elements 5.30.00 software ( Nikon Instruments Inc.).

### 4.6. Cell Cycle Assay

HASMCs were collected and washed twice with phosphate-buffered saline (PBS), followed by centrifugation to remove the supernatant. For fixation and permeabilization, cells (1.0 × 10^6^) were treated with 70% ethanol and incubated at 4 °C for 24 h. Subsequently, the cells were resuspended in PI/RNase staining buffer and incubated in the dark at room temperature for 30 min. Cell cycle distribution was then analyzed using a Coulter-XL flow cytometer (Beckman Coulter, Brea, California, USA) and ModFit LT 3.0 software (Verity Software House, Topsham, ME, USA).

### 4.7. Transwell Migration Assay

Cell migration was assessed using 24-well Transwell Permeable Supports. Briefly, HASMCs subjected to mechanical stretch were trypsinized and resuspended to a density of 3 × 10^4^ cells in 100 µL of medium containing 0.25% FBS. This cell suspension (100 µL) was loaded into the upper chamber, while the lower chamber was filled with 600 µL of DMEM supplemented with 10% FBS. After 12 h of incubation at 37 °C under 5% CO_2_, the filter membrane was fixed with 4% paraformaldehyde for 10 min. Non-migrated cells on the upper surface were gently removed with a cotton swab, and the migrated cells on the lower surface were stained with 0.2% crystal violet in 10% methanol for 30 min. The chambers were then thoroughly rinsed with water, and migrated cells were imaged under a light microscope (MATEO TL RUO, Leica Microsystems, Germany). Each experiment was repeated at least three times, and migration ability was quantified by counting the number of migrated cells.

### 4.8. Wound Healing Assay

HASMCs or PKM2-KO HASMCs were seeded in 6-well plates at a density of 1.5 × 10^5^ cells per well and treated with or without sodium lactate (NALA) for 48 h. Upon reaching confluence, a uniform linear wound was created in the cell monolayer using a 200 μL sterile pipette tip. The wells were then gently washed with fresh medium to remove detached cells and debris. Wound closure was monitored at 0, 12, and 24 h post-scratching using a Leica TL light microscope (MATEO TL RUO, Leica Microsystems) equipped with a digital camera. The extent of cell migration was quantified by measuring the reduction in the wound area over time.

### 4.9. Crosslinking Assay

For PKM2 crosslinking, HASMCs were collected and washed three times with PBS (pH 8.0). The cells were then incubated in PBS (pH 8.0) containing 2 mM disuccinimidyl suberate (DSS) for 30 min at room temperature. The reaction was quenched by adding 20 mM Tris-HCl (pH 8.0) and incubating for 15 min. After centrifugation at 1000× *g* for 5 min, the cell pellets were lysed in RIPA buffer for subsequent protein extraction and Western blot analysis.

### 4.10. Nuclear and Cytoplasmic Protein Extraction

The subcellular localization of PKM2 was assessed by separating nuclear and cytoplasmic fractions using a Nuclear and Cytoplasmic Protein Extraction Kit according to the manufacturer’s protocol. PKM2 nuclear translocation was then evaluated by comparing the relative protein levels in the nuclear and cytoplasmic fractions via Western blotting.

### 4.11. Immunoprecipitation Assay

Immunoprecipitation was performed using an rProtein A/G Magnetic IP/Co-IP Kit. Briefly, 500 μg of cell lysate was pre-incubated with 2 μg of an anti-L-lactyl lysine antibody for 30 min at room temperature. The mixture was then incubated with rProtein A/G magnetic beads for 3 h at room temperature to capture the antigen–antibody complexes. The bound proteins were eluted by heating the beads in reducing SDS-PAGE loading buffer, and the eluted samples were subsequently subjected to Western blot analysis using an anti-rabbit IgG for IP detection.

### 4.12. Western Blotting

HASMCs were collected and lysed in RIPA buffer supplemented with PMSF. Protein concentration was determined using a BCA assay. For Western blot analysis, proteins were separated by SDS-PAGE and electrophoretically transferred to PVDF membranes. After blocking with 5% BSA for 2 h at room temperature, the membranes were incubated with specific primary antibodies at recommended dilutions overnight at 4 °C. The following day, membranes were incubated with horseradish peroxidase (HRP)-conjugated secondary antibodies (1:5000 dilution) for 1 h at room temperature, followed by washing with PBST. Immunoreactive bands were visualized using enhanced HyperSignal ECL substrate on an ImageQuant 800 biomolecular imager (Cytiva, Marlborough, Massachusetts, USA). Band intensity was quantified using ImageQuant 800 ver 1.2 software (Cytiva), and GAPDH was used as a loading control.

### 4.13. Immunofluorescence Staining

HASMCs treated with 20 mM NALA were fixed with 4% paraformaldehyde for 15 min and permeabilized with 0.5% Triton X-100 for 20 min at room temperature. To minimize nonspecific binding, cells were blocked with a solution containing 0.3% bovine serum albumin and 1% Triton X-100 for 1 h at room temperature. Subsequently, the cells were incubated overnight at 4 °C with an anti-PKM2 antibody (1:200 dilution). After washing, samples were incubated with an Alexa Fluor 594-conjugated secondary antibody (1:1000 dilution) for 1 h at room temperature. Nuclei were counterstained with DAPI in the dark. Fluorescence images were acquired using a Nikon ECLIPSE Ti2 microscope (Nikon Instruments Inc.), and a fixed threshold was applied uniformly to all images to exclude background signal during analysis.

### 4.14. OCR and ECAR Detection

ECAR (indicative of glycolysis) and OCR (indicative of oxidative phosphorylation) were measured using an XF96 Extracellular Flux Analyzer (Seahorse Biosciences, North Billerica, Massachusetts, USA) with the Agilent Seahorse XF Glycolytic Rate Assay Kit and the Agilent Seahorse XF Cell Mito Stress Test Kit, respectively. Raw data were processed using Seahorse Wave 2.6.4 software (Agilent Technologies, Santa Clara, California, USA). For mechanical stretch experiments, HASMCs were seeded into Seahorse XF96 cell culture microplates at a density of 4 × 10^4^ cells per well after stretch stimulation. Following overnight incubation (37 °C, 5% CO_2_), the culture medium was replaced with 120 µL of XF assay medium. For sodium lactate (NALA) treatment, cells were seeded at 1 × 10^4^ cells per well and incubated with 20 mM NALA for 48 h prior to the assay. For the mitochondrial stress test, the following compounds were sequentially injected into each well: 1 µM oligomycin, 1 µM carbonyl cyanide-p-trifluoromethoxyphenylhydrazone (FCCP), and 0.5 µM rotenone/antimycin A. Basal OCR was defined as the measurement taken prior to oligomycin injection. Maximal OCR was determined as the peak rate following FCCP injection. For the glycolytic rate assay, 10 mM glucose, 1.0 µM oligomycin A, and 50 mM 2-deoxy-D-glucose (2-DG) were sequentially injected. Basal glycolysis was calculated as the difference between the last measurement prior to oligomycin injection and the non-glycolytic acidification rate. The maximum ECAR rate was determined as the peak rate following oligomycin injection. All ECAR and OCR values were normalized to the cell count in each corresponding well.

### 4.15. Statistical Analysis

Data analysis was conducted using GraphPad Prism software (8.3.0.). The results are presented as the mean ± SM of three independent biological experiments. The differences between two groups were analyzed using Student’s *t*-test. For multiple comparisons of more than two normally distributed groups, one-way ANOVA with Dunnett’s post-test was used. *p* < 0.05 was considered statistically significant.

## 5. Conclusions

Our study unveils a complete pathway that cyclic mechanical stress promotes VSMC phenotypic switching via PKM2-mediated glycolysis, while lactate serves as an endogenous feedback regulator by enforcing PKM2 tetramerization. This newly identified “lactate-PKM2 axis” not only deepens our understanding of vascular mechanobiology but also presents a promising molecular target for developing novel interventions to protect astronaut cardiovascular health during spaceflight.

## Data Availability

The original contributions presented in this study are included in the article/Supplementary Material. Further inquiries can be directed to the corresponding authors.

## Supplementary Materials

The following supporting information can be downloaded at: https://www.mdpi.com/article/10.3390/ijms27073298/s1.
