# Supplementary material for: Mechanisms of Mechanical Stress-Induced Vascular Remodeling via the Lactate-PKM2 Axis and Implications for Microgravity Adaptation
Source: Int J Mol Sci. 2026 Apr 5;27(7):3298. doi: 10.3390/ijms27073298 (PMC13073293; doi:10.3390/ijms27073298)
Supplement: Supplementary file 1 [file ijms-27-03298-s001.zip › Supplementary Table S2. PKM2 targeting siRNAs sequence.docx]

| Name | Sequence |
| --- | --- |
| siRNA-PKM21 | AGGGAAAGAACATCAAGATTA |
| siRNA-PKM21 | TGGATAACGCCTACATGGAAA |

**Supplementary table 2. PKM2 targeting siRNAs sequence**
